# Molecular Evolutionary Analyses of the RNA-Dependent RNA Polymerase (*RdRp*) Region and *VP1* Gene in Sapovirus GI.1 and GI.2

**DOI:** 10.3390/microorganisms13020322

**Published:** 2025-02-01

**Authors:** Fuminori Mizukoshi, Ryusuke Kimura, Tatsuya Shirai, Asumi Hirata-Saito, Eri Hiraishi, Kosuke Murakami, Yen Hai Doan, Hiroyuki Tsukagoshi, Nobuhiro Saruki, Takeshi Tsugawa, Kana Kidera, Yoshiyuki Suzuki, Naomi Sakon, Kazuhiko Katayama, Tsutomu Kageyama, Akihide Ryo, Hirokazu Kimura

**Affiliations:** 1 Department of Virology III, National Institute of Infectious Diseases, Musashimurayama-shi 208-0011, Japan; shirai@niid.go.jp (T.S.); aryo@niid.go.jp (A.R.); 2 Department of Bacteriology, Graduate School of Medicine, Gunma University, Maebashi-shi 371-8511, Japan; m2220015@gunma-u.ac.jp; 3 Advanced Medical Science Research Center, Gunma Paz University, Takasaki-shi 370-0006, Japan; 4 Department of Microbiology, Tochigi Prefectural Institute of Public Health and Environmental Science, Utsunomiya-shi 329-1196, Japan; saitoa01@pref.tochigi.lg.jp; 5 Department of Health Science, Gunma Paz University Graduate School of Health Sciences, Takasaki-shi 370-0006, Japan; er.hiraishi@gmail.com; 6 Center for Emergency Preparedness and Response, National Institute of Infectious Diseases, Shinjuku-ku 162-8640, Japan; ko-mura@niid.go.jp; 7 Center for Emergency Preparedness and Response, National Institute of Infectious Diseases, Musashimurayama-shi 208-0011, Japan; yendoan@niid.go.jp (Y.H.D.); tkage@niid.go.jp (T.K.); 8 Gunma Prefectural Institute of Public Health and Environmental Sciences, Maebashi-shi 371-0052, Japan; tsuka-hiro@pref.gunma.lg.jp (H.T.); saruki-n@pref.gunma.lg.jp (N.S.); 9 Department of Pediatrics, Sapporo Medical University School of Medicine, Sapporo-shi 060-8543, Japan; tsugawat@sapmed.ac.jp; 10 Laboratory of Viral Infection Control, Ōmura Satoshi Memorial Institute, Graduate School of Infection Control Sciences, Kitasato University, 5-9-1, Shirogane, Minato-ku 108-8641, Japan; kidera.kana@st.kitasato-u.ac.jp (K.K.); katayama@lisci.kitasato-u.ac.jp (K.K.); 11 Division of Biological Science, Department of Information and Basic Science, Graduate School of Sciences, Nagoya City University, Nagoya-shi 467-8501, Japan; yossuzuk@nsc.nagoya-cu.ac.jp; 12 Department of Microbiology, Osaka Institute of Public Health, Osaka 537-0025, Japan; nsakon@iph.osaka.jp

**Keywords:** human sapovirus, molecular evolutionary analyses, RNA-dependent RNA polymerase (*RdRp*), capsid (*VP1*)

## Abstract

Human sapovirus (HuSaV) is a significant cause of gastroenteritis. This study aims to analyze the evolutionary dynamics of the RNA-dependent RNA polymerase (*RdRp*) and capsid (*VP1*) genes of the HuSaV GI.1 and GI.2 genotypes between 1976 and 2020. Using bioinformatics tools such as the Bayesian phylogenetics software BEAST 2 package (v.2.7.6), we constructed time-scale evolutionary trees based on the gene sequences. Most of the recent common ancestors (MRCAs) of the *RdRp* region and *VP1* gene in the present HuSaV GI.1 diverged around 1930 and 1933, respectively. The trees of the HuSaV GI.1 *RdRp* region and *VP1* gene were divided into two clusters. Further, the MRCAs of the *RdRp* region and *VP1* gene in HuSaV GI.2 diverged in 1960 and 1943, respectively. The evolutionary rates were higher for *VP1* gene in HuSaV GI.1 than that in HuSaV GI.2, furthermore, were higher in GI.1 Cluster B than GI.1 Cluster A. In addition, a steep increase was observed in the time-scaled genome population size of the HuSaV GI.1 Cluster B. These results indicate that the HuSaV GI.1 Cluster B may be evolving more actively than other genotypes. The conformational B-cell epitopes were predicted with a higher probability in *RdRp* for GI.1 and in *VP1* for GI.2, respectively. These results suggest that the *RdRp* region and *VP1* gene in HuSaV GI.1 and GI.2 evolved uniquely. These findings suggest unique evolutionary patterns in the *RdRp* region and *VP1* gene of HuSaV GI.1 and GI.2, emphasizing the need for a ‘One Health’ approach to better understand and combat this pathogen.

## 1. Introduction

Sapovirus (SaV) is a non-enveloped, positive-sense, single-stranded RNA virus that belongs to the Caliciviridae family, which also includes norovirus (NoV) [1]. Various mammals, including humans, pigs, dogs, bats, minks, sea lions, and rats are susceptible to SaV [1,2,3]. Currently, SaV is classified into nineteen genogroups (GI to GXIX). Four of these genogroups, GI, GII, GIV, and GV, can infect humans and cause clinical disease [1,4]. Moreover, human SaV (HuSaV) is divided into 18 genotypes: GI.1 to GI.7, GII.1 to GII.8, GIV.1, GV.1, and GV.2 [1,5,6]. Molecular epidemiological data on HuSaV infections in humans suggest that two genotypes, GI.1 and GI.2, are frequently detected worldwide [4,7,8,9,10].

HuSaV is a causative agent of viral acute gastroenteritis in outbreaks and sporadic cases worldwide [1,4]. The spread of HuSaV usually occurs via the fecal–oral route from contaminated foods and/or via person-to-person transmission. Major clinical symptoms of HuSaV include diarrhea, vomiting nausea, chills, and abdominal cramps. Furthermore, a significant exacerbation of the HuSaV infection occasionally leads to hospitalization [11]. However, at present, effective vaccines and antiviral agents are not available for the control and prevention of HuSaV infections [12]. Therefore, this agent may be a public health concern.

The SaV genome is a positive-sense, single-stranded RNA with an approximately 7.1 to 7.7 kb nucleotide sequence and is composed of two open reading frames (ORF), ORF1 and ORF2 [1]. ORF1 encodes the nonstructural proteins, including RNA-dependent RNA polymerase (RdRp) and the major structural protein, capsid (VP1). ORF2 encodes the minor structural protein, VP2 [1]. The *VP1* gene is more diverse than the *RdRp* gene and is an important region for genotyping [1]. Moreover, similar to NoV, recombinant strains between the *RdRp* gene and *VP1* gene have been reported [1]. Therefore, to understand the characteristics of the SaV genome, it is important to analyze these two major genes, the *RdRp* gene and *VP1* gene, in detail.

It has been recognized that the *RdRp* region and *VP1* gene of NoV have evolved in a co-dependent manner [13,14]. Although most studies have focused on the molecular evolution of NoVs, that of HuSaV is not well known. To gain insight into this theme, we performed a comprehensive molecular analysis of the *RdRp* region and *VP1* gene of the HuSaV GI.1 and GI.2 detected globally between 1976–2020.

## 2. Materials and Methods

### 2.1. Strains in This Study

To analyze the molecular evolution of HuSaV GI.1 and GI.2, the complete genome sequences of SaV were downloaded from NCBI Virus [https://www.ncbi.nlm.nih.gov/labs/virus/vssi/#/] (last accessed on 2 November 2023) by being searched as the query “Sapovirus, taxid:95341”. To begin with, 5750 strains were collected from the database search. These strains were classified into genotypes using the Norovirus Typing Tool (Ver.2.0) and were confirmed to be defined as SaV GI.1 and GI.2 [15]. Of these, strains with an ambiguous sequence (e.g., N, Y, R, and V) or an unclear year of collection or area were omitted from the dataset. Moreover, sequences with 100% identity were excluded from the dataset. Finally, 113 and 57 strains belonging to HuSaV GI.1 and GI.2 remained and were used to analyze the molecular evolution of the *VP1* gene. On the other hand, 86 and 57 strains belonging to SaV GI.1 and GI.2 were obtained and used to analyze the molecular evolution of the *RdRp* region. Detailed information on the strains used in this study are shown in Supplementary Table S1. Distributions of these genotypes during the investigation periods (1976–2020) are shown in Supplementary Figure S1.

### 2.2. Maximum Likelihood (ML)

Multiple alignments for these sequences were performed using MAFFT, version 7.520 [16]. The ML phylogenetic analysis was performed using IQ-TREE, version 2.2.2.6 with ultrafast bootstrap test parameters and the SH-like approximate likelihood ratio test [17]. The best-fitting substitution models were automatically selected using ModelFinder within IQ-TREE. FigTree, version 1.4, was used to visualize the resultant trees.

### 2.3. Time-Scaled Phylogenetic Analyses and Estimation of Evolutionary Rate

To evaluate the molecular evolution of the present strains, phylogenetic trees of the HuSaV *RdRp* region and *VP1* gene were constructed using the Bayesian Markov chain Monte Carlo (MCMC) method in the BEAST package (v.2.7.6) [18]. First, suitable substitution models of each dataset were estimated and determined by the jModelTest2 program [19]. Second, nested sampling was performed to evaluate the best combination from the six clock models (strict clock, random local clock, optimized relaxed clock, relaxed clock exponential, relaxed clock log normal, and fast relaxed clock log normal) and the two prior tree models (coalescent constant population, and coalescent exponential population) [20]. Based on the obtained suitable combinations of these models, listed in Supplementary Table S2, MCMC trees were constructed using the Beast2 software (v.2.7.6). The MCMC chains consisted of 300,000,000 steps, with sampling at every 1000 steps. To confirm convergence, Tracer, version 1.7.2, was used to evaluate effective sample sizes (ESS); values above 200 were accepted. After discarding the first 10% of the MCMC chain as burn-in, the maximum clade credibility tree was produced by TreeAnnotator, version 2.7.6, in the BEAST package. The Bayesian MCMC phylogenetic tree was illustrated by FigTree, version 1.4; 95% of the highest posterior densities (HPDs) of all internal nodes were computed.

In addition, the molecular evolutionary rates were estimated using suitable models selected for each dataset, as described above. Statistical analyses were performed using the Kruskal–Wallis test for GraphPad Prism 7 (GraphPad Software, La Jolla, CA, USA). A significant difference was defined as *p* < 0.0001.

### 2.4. Bayesian Skyline Plot (BSP) Analyses

To evaluate the phylodynamics of the HuSaV strains and each cluster, the effective population size of the *RdRp* region and *VP1* gene were calculated using BSP analysis with BEAST, v2.7.6 [18]. The best substitution combinations of these models were selected, as described above. The best clock model in combination with the prior tree models (coalescent Bayesian skyline) was selected from the models (strict clock, random local clock, optimized relaxed clock, relaxed clock exponential, relaxed clock log normal, and fast relaxed clock log normal), as described above. The obtained suitable models are listed in Supplementary Table S2. The MCMC chains were run for 300,000,000 steps, with sampling at every 1000 steps. The visualization of the BSPs with 95% of the HPDs were performed using Tracer, v1.7.2 [21].

### 2.5. Phylogenetic Distance Analyses

The phylogenetic distances (patristic distances) between the HuSaV strains and each cluster were analyzed and calculated from the above-mentioned ML tree using the Patristic program [22]. A violin plot was constructed using Orange DATA MINING, version 3.35 [23]. Statistical analyses were performed using the unpaired t test with Welch’s correction for GraphPad Prism 7 (GraphPad Software, La Jolla, CA, USA). A significant difference was defined as *p* < 0.0001.

### 2.6. Similarity Plot Analyses

The nucleotide similarity of each sequence, using SimPlot program 3.5.1, was calculated to clarify the relationships among the aligned nucleotide sequences of the HuSaV *RdRp* region and *VP1* gene [24]. Two strains (GI.1: MN794208/HKG/1977 and GI.2: MN794205/GUF/1978) were used as reference sequences. The similarity was examined using the Kimura two-parameter method, with a window size of 200 nucleotides and a step size of 20 nucleotides.

### 2.7. Construction of the 3D Structure of RdRp and VP1 Proteins

To compare the RdRp and VP1 protein structures among the subtypes, three-dimensional (3D) structural models of these proteins were predicted using LocalColabFold, version 1.5.3, installed on a local computer [25]. Structural models of the HuSaV RdRp and VP1 proteins were constructed for representative strains from each genotype (GI1: MN794208[1977], LC671561[2020/Cluster A], and OP654153[2020/Cluster B]; and GI2: MN794205[1978], and MN510438[2018]). First, the multiple sequence alignment files (a3m files) were constructed on a local computer, using uniref30(2302) as the uniref database, PDB100(230517) as the template database, and colabfold_envdb(202108) as the environmental sequence database. Second, the following flags were used for the structure prediction: “--amber”; “--templates”; and “--use-gpu-relax”. The number of prediction recycles was 30. Of the 5 prediction models created with LocalColabfold for each sequence, the best model was determined by considering parameters such as the predicted local distance difference test (pLDDT), template modeling (TM)-score, and root mean square deviation (RMSD). Lastly, these final models were visualized by UCSF ChimeraX, version 1.7.1 [26].

### 2.8. Conformational B-Cell Epitope Prediction

The conformational B-cell epitopes of the constructed RdRp and VP1 protein models were evaluated using the following five methods: DiscoTope 3.0 (higher confidence:1.50, recall up to ~30%) [27]; ElliPro (cutoff values of 0.5) [28]; epitope3D [29]; SEPPA 3.0 (cutoff values of 0.089) [30]; and SEMA (cutoff values of 0.76) [31]. Amino acid residues predicted by four or more of these methods were determined as conformational B-cell epitopes. These predicted B-cell epitopes were mapped and colored on each model using UCSF ChimeraX, version 1.7.1 [26].

The number of methods predicted as epitopes were visualized using heat maps. The heat maps were constructed with Orange DATA MINING, version 3.35 [23].

### 2.9. Selective Pressure Analyses

To assume the selective pressure sites for the *RdRp* region and *VP1* gene, the non-synonymous (*dN*) and synonymous (*dS*) substitution rates at each amino acid site were estimated using Datamonkey [32]. The following three algorithms were used to identify negatively selected sites: single-likelihood ancestor counting (SLAC) [33]; fixed effects likelihood (FEL) [33]; and fast unconstrained Bayesian approximation (FUBAR) [34]. Next, in addition to the above three methods (SLAC, FEL, and FUBAR), the mixed effects model of evolution (MEME) [35] method was used to detect positively selected sites. The significance level was set at *p* < 0.05 for SLAC, FEL, and MEME. Evidence of selective pressure for FUBAR was supported by a posterior probability > 0.9.

## 3. Results

### 3.1. The Phylogeny of the RdRp Region and VP1 Gene in HuSaV Genogroups

The phylogenetic trees (ML method) of the various HuSaV genogroups (GI.1 and GI.2, plus GI.6, GII, GIV, GV, and porcine SaV GIII) based on the full-length nucleotides of the *RdRp* region and *VP1* gene are shown in Supplementary Figure S2. The trees of *RdRp* and *VP1* showed nearly similar tree shapes, suggesting that these genes may have co-evolved within the SaV genogroups. However, HuSaV GI.6 was located on a different branch, predicting that the recombination between the *RdRp* region and *VP1* gene may have occurred previously. Moreover, the sequences of HuSaV GI.1 and GI.2 were closely related on the *RdRp* region and *VP1* gene, respectively.

The ML trees of each genotype (GI.1 or GI.2) on the *RdRp*/*VP1* sequences were constructed, respectively (Supplementary Figure S3A–D). The trees of the HuSaV GI.1 *RdRp* region and *VP1* gene were subdivided into two clusters, defined as Cluster A and Cluster B in this study. Distributions of these clusters are shown in Supplementary Figure S1.

### 3.2. Time-Scaled Phylogeny

**Figure 1:**
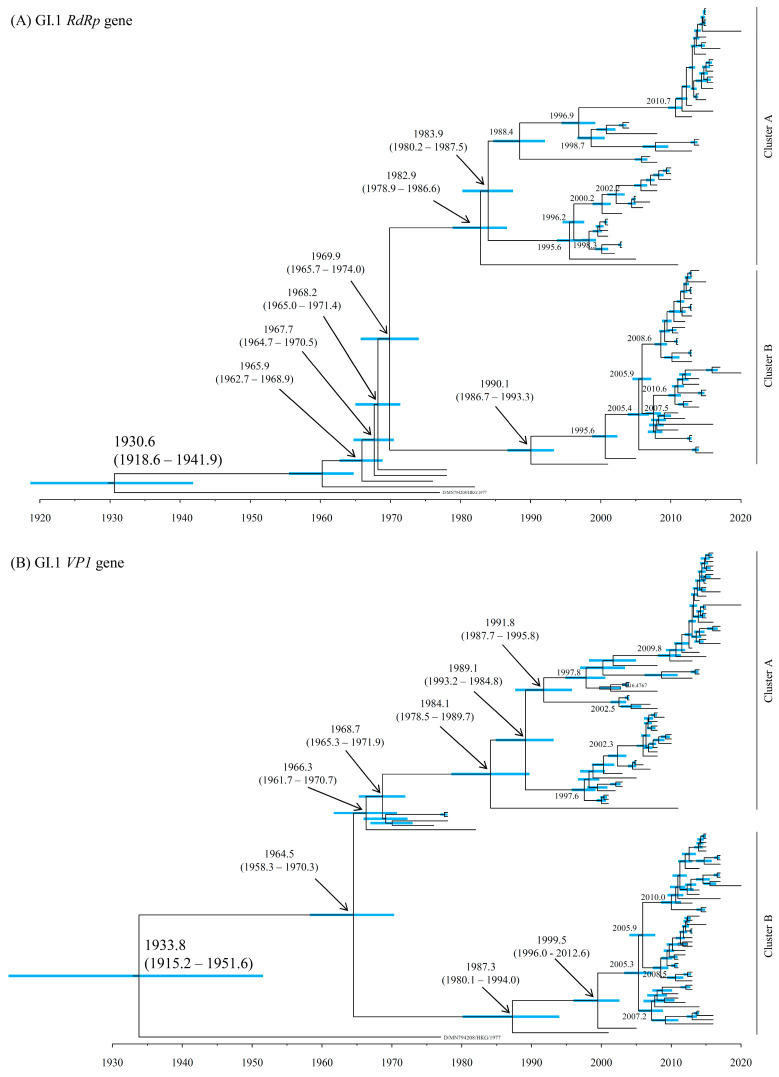

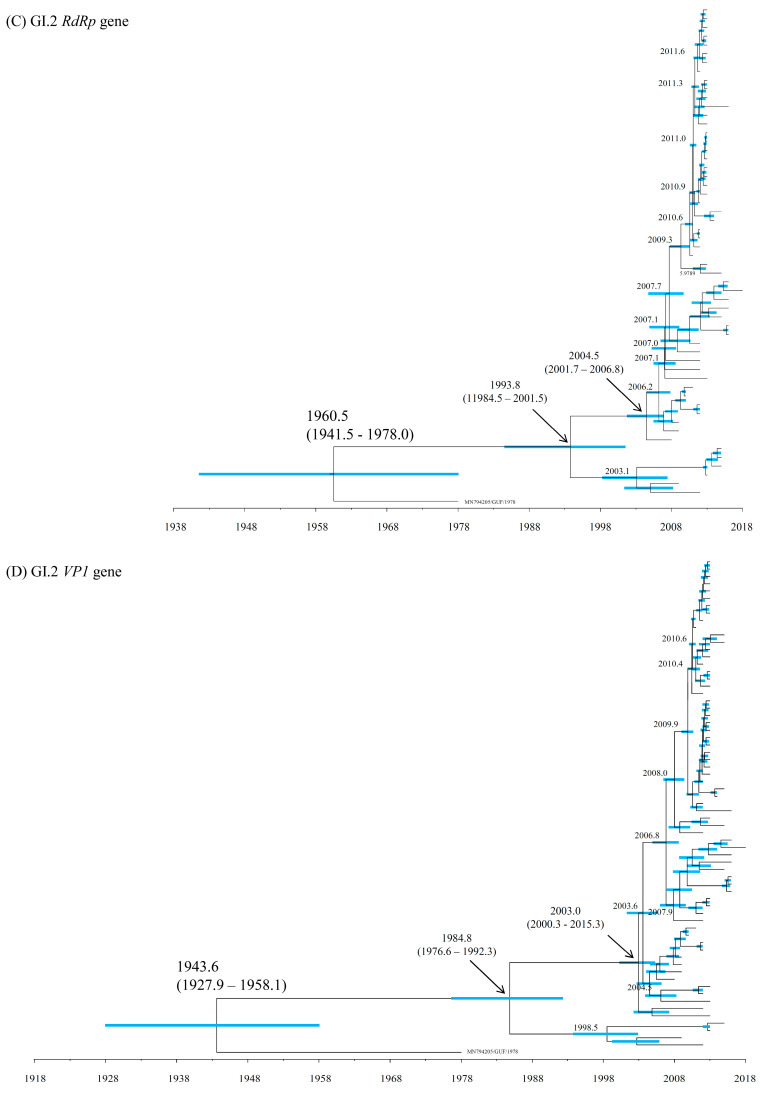
Time-scaled phylogenetic tree of the HuSaV GI.1 constructed by the Bayesian MCMC method: *RdRp* region (**A**); and *VP1* gene (**B**). Time-scaled phylogenetic tree of the HuSaV GI.2 constructed by the Bayesian MCMC method: *RdRp* region (**C**); and *VP1* gene (**D**). The horizontal axis represents time (years). Blue bars indicate the 95% HPD for a branched year.

To estimate the evolution of the *RdRp* region and *VP1* gene in HuSaV GI.1 and GI.2, time-scale phylogenetic trees were constructed based on these sequences (Figure 1A–D). Most recent common ancestors (MRCAs) of the *RdRp* region and *VP1* gene in HuSaV GI.1 diverged around 1930.6 (mean; 95% HPDs, 1918.6–1941.9) and 1933.8 (mean; 95% HPDs, 1915.2–1951.6), respectively (Figure 1A,B). Subsequently, these genes of HuSaV GI.1 were further divided into two clusters, Cluster A and Cluster B. The common ancestors of the *RdRp* region of Cluster A and Cluster B diverged around 1982.9 and 1990.1, respectively. On the other hand, the common ancestors of the *VP1* gene of Cluster A and Cluster B diverged around 1984.1 and 1987.3, respectively. Further, the MRCAs of the *RdRp* region and *VP1* gene in HuSaV GI.2 diverged in 1960.5 (mean; 95% HPDs, 1941.5–1978.0) and 1943.6 (mean; 95% HPDs, 1927.9–1958.1), respectively (Figure 1C,D). The common ancestors of the current HuSaV GI.2 strains detected in 2008–2018 diverged in 1993.8 (*RdRp* region) and 1984.8 (*VP1* gene), respectively.

Next, to estimate the ancestors of various genogroups of SaV, time-scale phylogenetic trees were constructed based on the sequences of HuSaV GI.1 and GI.2, together with HuSaV GI.6, GII, GIV, GV, and porcine SaV GIII (Supplementary Figure S4). As a result, the *RdRp* region and *VP1* gene of the HuSaV genogroups (GI, GII, GIV, and GV) diverged from those of the ancestor porcine SaV GIII in 546.6 BC (mean; 95% HPDs, 1269.2 BC–108.4 BC) and 929.7 BC (mean; 95% HPDs, 1668.5 BC–242.8 BC), respectively. The *RdRp* region of HuSaV GI.1 and GI.2 diverged at 1686.6 (mean; 95% HPDs, 1630.0–1737.6). On the other hand, the common ancestors of the HuSaV GI.1 and GI.2 *VP1* genes divided in 1738.2 (mean; 95% HPDs, 1696.1–1778.1). However, because the phylogenetic trees of the various genogroups were constructed with the alignment data of the different nucleotide lengths, this may reflect on the accuracy of the results and this bias may limit the phylogenetic trees of various genogroups.

### 3.3. Evolutionary Rates

**Figure 2:**
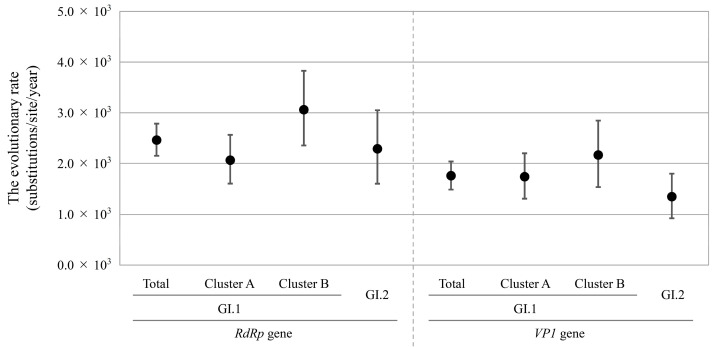
Evolutionary rates of each sequence in the HuSaV of *RdRp* region and *VP1* gene. The y-axis represents the evolutionary rate (substitutions/site/year), and the x-axis indicates each genotype and cluster. The black circles indicate the mean and the bars indicate the intervals of 95% HPD. The detailed evolutionary rates are shown in Supplementary Table S3.

The evolutionary rates of the *RdRp* region and *VP1* gene in HuSaV GI.1 and GI.2 are shown in Figure 2 and Supplementary Table S3. In each *RdRp* region and *VP1* gene, there were significant differences in all combinations of genotypes and clusters.

The evolutionary rate of the *RdRp* region was higher for the total HuSaV GI.1 (2.461 × 10^−3^ substitutions/site/year) than the total HuSaV GI.2 (2.289 × 10^−3^ substitutions/site/year). Similarly, the mean evolutionary rate of the *VP1* gene in the total HuSaV GI.1 strains (1.763 × 10^−3^ substitutions/site/year) was higher than that in the HuSaV GI.2 (1.350 × 10^−3^ substitutions/site/year).

Comparing HuSaV GI.1 Cluster A and Cluster B, the evolutionary rates of the *RdRp* region and *VP1* gene were higher in Cluster B (3.059 and 2.168 × 10^−3^ substitutions/site/year, respectively) than in Cluster A (2.065 × 10^−3^ and 1.745 × 10^−3^ substitutions/site/year, respectively).

These results indicate that, comparing the evolutionary rates among the genotypes and clusters, that of GI.1 was faster than that of GI.2, and that of GI.1 Cluster B was the most rapid.

### 3.4. Phylodynamic Using the BSP Method

**Figure 3:**
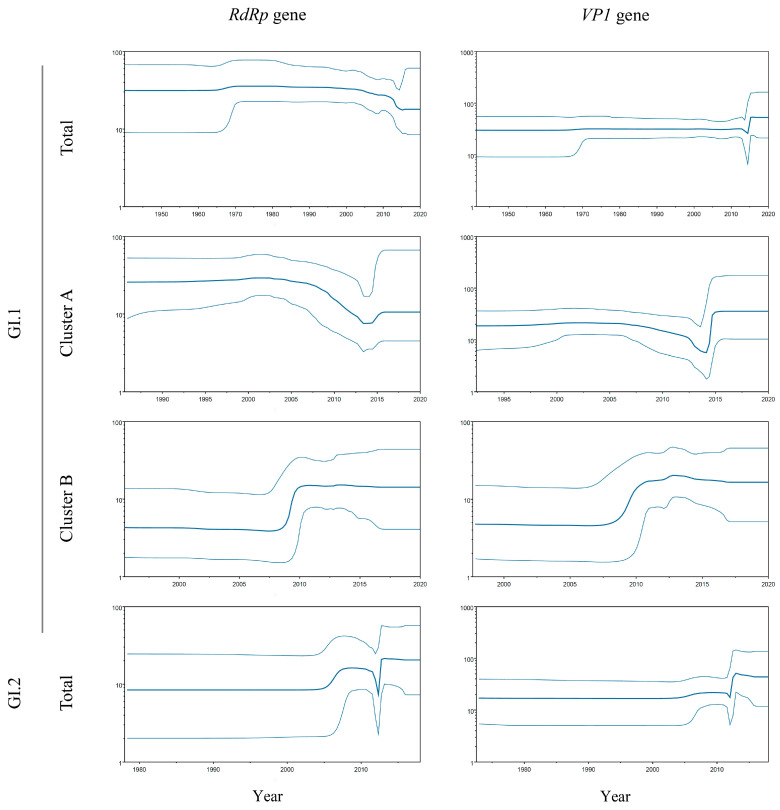
Phylodynamics of the *RdRp* region and *VP1* genes in HuSaV GI.1 and GI.2 determined using BSP analysis. The y-axis represents the effective population size on the logarithmic scale and the x-axis indicates the time in years. The thick line (blue) shows the median value over time. The intervals with the HPDs (95%) are shown by thin lines (blue).

The phylodynamics of HuSaV GI.1 and GI.2 in the *RdRp* region and *VP1* gene were estimated and the effective population sizes (EPS) are shown in Figure 3. Until approximately the 2000s, the genome population sizes of both the *RdRp* region and *VP1* gene remained constant. However, significant fluctuations in the EPS were observed around 2010.

In particular, the EPS of the *RdRp* region and *VP1* gene in HuSaV GI.1 Cluster B rapidly increased before 2010 and then remained constant. On the other hand, the EPS of the *VP1* gene in Cluster A slightly increased around 2010 but that in the *RdRp* gradually decreased after around 2015. These results suggest that HuSaV GI.1 Cluster B has become more adapted to humans.

### 3.5. Phylogenetic Distances

**Figure 4:**
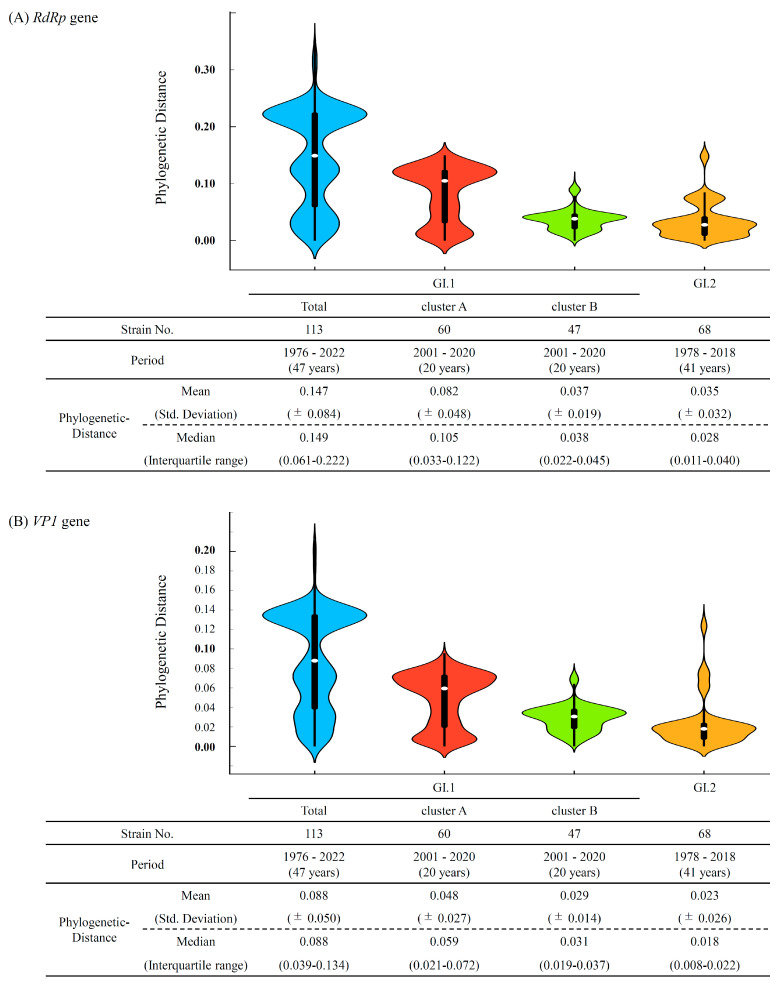
Phylogenetic distances of the *RdRp* region (**A**); and *VP1* gene (**B**) in HuSaV GI.1 and GI.2, illustrated by violin plots. The width of the violin plot represents the kernel density, indicating the distribution shape of the data. Total HuSaV GI.1, GI.1 Cluster A, GI.1 Cluster B, and HuSaV GI.2 are illustrated in light blue, red, green, and gold. The central box plot (black) and white dots represent the interquartile range and the median, respectively. The whiskers from the box plots represent the data intervals. The detailed statistical data are shown below the violin plots. There were significant differences in all combinations of genotypes and clusters (unpaired *t* test; *p* < 0.0001).

The genetic divergence of the *RdRp* region and *VP1* gene was assessed by their phylogenetic distances and distribution (Figure 4A,B). In both the *RdRp* region and *VP1* gene, there were significant differences in all combinations of genotypes and clusters.

The phylogenetic distance values of the *RdRp* region in HuSaV GI.1 Cluster A, Cluster B, and in the GI.2 strains were 0.082 ± 0.048, 0.037 ± 0.019, and 0.035± 0.032 (mean ± SD). Moreover, the phylogenetic distance values of the *VP1* gene in HuSaV GI.1 Cluster A, Cluster B, and in the GI.2 strains were 0.048 ± 0.027, 0.029 ± 0.014, and 0.023 ± 0.026 (mean ± standard deviation, SD). In both the *RdRp* region and *VP1* gene, the branches of the HuSaV GI.1 Cluster A were longer than those of the others (*p* < 0.0001).

These results indicate that the genetic divergence of GI.2 was more highly conservative than that of the other, and that GI.1 Cluster A has a statistically greater genetic divergence than GI.1 Cluster B.

### 3.6. Similarity Plot Analysis

**Figure 5:**
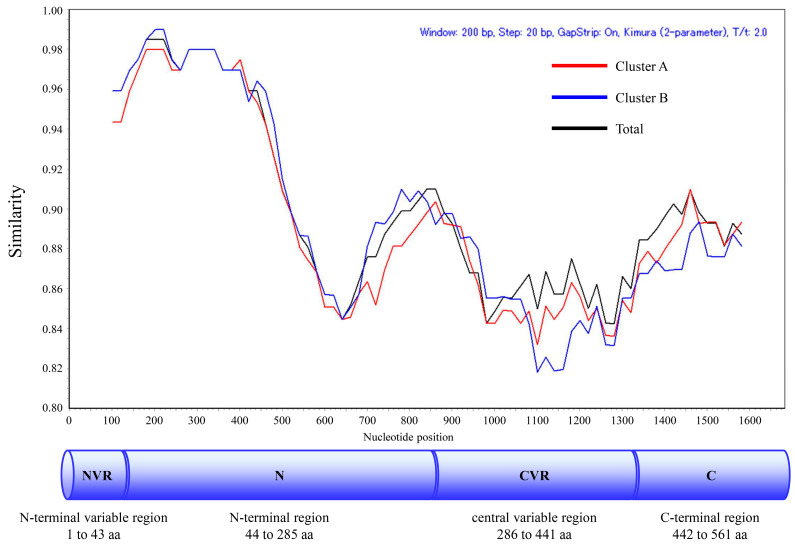
Similarity plot analysis of the *VP1* gene in HuSaV GI.1. Nucleotide similarity to the prototype strain (GenBank accession no. MN794208, collected in 1977) was calculated using SimPlot analysis. Total HuSaV GI.1, GI.1 Cluster A, and GI.1 Cluster B are illustrated in black, red, and blue. Nucleotide position numbers correspond to the *VP1* gene in the prototype strain. The positions of each subunit are shown below the graph [1].

To understand the genetic diversity of intra-genotypes, a similarity plot analysis based on the nucleotide sequences of the *RdRp* region and *VP1* gene was conducted and is shown in Figure 5 and in Supplementary Figure S5. The results show that the S domain (N-terminal region) and P2 domain (central variable region) in the *VP1* gene of the HuSaV GI.1 total strain, Cluster A, and Cluster B, are characterized by lower similarities (approximately 85% and 82%, respectively), indicating that high divergence sites are localized in the *VP1* gene. On the other hand, the similarity of the entire *VP1* gene in HuSaV GI.2 strains was slightly low (80–90%).

### 3.7. 3D Mapping of the Conformational Epitopes in the RdRp Dimer and VP1 Trimer Proteins

**Figure 6:**
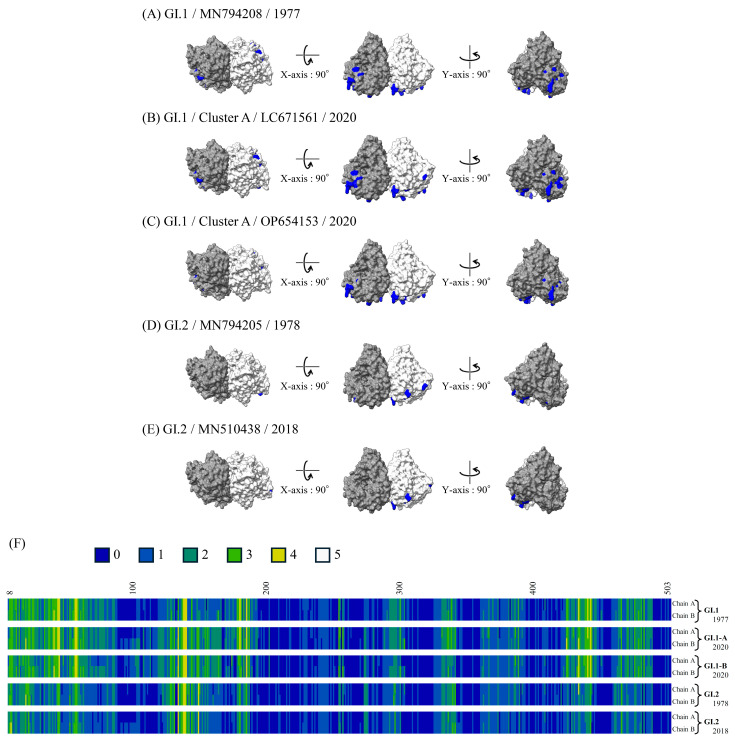
Structural models of the RdRp dimer proteins and mapping of predicted conformational epitopes. Chains A and B are colored by gray and light gray, respectively. The predicted conformational epitopes are shown in blue. The strains are as follows: (**A**) GI.1 prototype strain collected in 1977 (MN794208); (**B**) GI.1 strain belonging to Cluster A collected in 2020 (LC671561); (**C**) GI.1 strain belonging to Cluster B collected in 2020 (OP654153); (**D**) GI.2 prototype strain collected in 1978 (MN794205); (**E**) GI.2 strain collected in 2018 (MN510438); and (**F**) the sites and the number of methods predicted as epitopes and visualized in heat maps. Amino acid residues predicted as conformational B-cell epitopes by 0, 1, 2, 3, 4, and 5 of the five methods are shown in dark blue, blue, dark green, green, yellow, and white.

**Figure 7:**
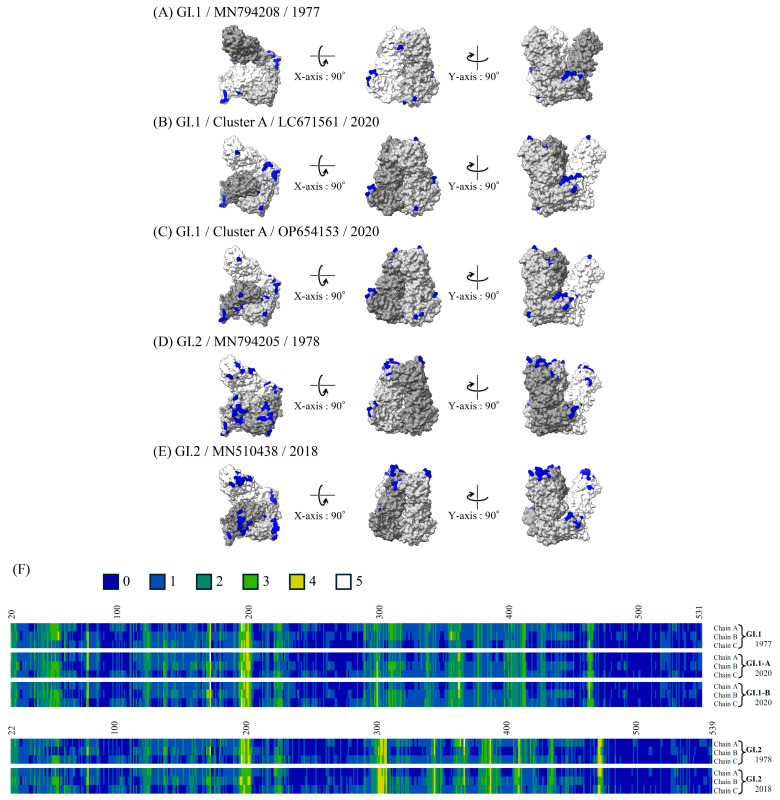
Structural models of the VP1 trimer proteins and mapping of predicted conformational epitopes. Chains A, B, and C are colored in dark gray, light gray, and white, respectively. The predicted conformational epitopes are shown in blue. The strains are as follows: (**A**) GI.1 prototype strain collected in 1977 (MN794208); (**B**) GI.1 strain belonging to Cluster A collected in 2020 (LC671561); (**C**) GI.1 strain belonging to Cluster B collected in 2020 (OP654153); (**D**) GI.2 prototype strain collected in 1978 (MN794205); (**E**) GI.2 strain collected in 2018 (MN510438); and (**F**) the sites and the number of methods predicted as epitopes and visualized in heat maps. Amino acid residues predicted as conformational B-cell epitopes by 0, 1, 2, 3, 4, and 5 of the five methods are shown in dark blue, blue, dark green, green, yellow, and white.

The predicted conformational epitopes mapped on the RdRp and VP1 protein structure models of the recent strains and ancient representative strains of each genotype are shown in Figure 6A and Figure 7A. To assess the reliability of the predicted sites, the number of methods predicted as epitopes are visualized in a heat map (Figure 6B and Figure 7B). Details of the sites predicted as the conformational epitopes are presented in Supplementary Tables S4 and S5.

More sites were predicted as epitopes in the RdRp protein of the GI.1 strains than in that of the GI.2 strains. Most of these predicted epitopes were located on the sides of the RdRp protein dimer in the GI.1 strains (residues aa42–59, 181,186, and 434). On the other hand, more sites predicted as epitopes (residues aa304–310, 347, 370, 390, 412, and 473–475) were found in the VP1 trimer protein of GI.2 than that of GI.1. Many of the predicted epitopes are located in the protruding position (mainly, P2 domain) of the trimer.

### 3.8. Negative and Positive Selection Sites

To determine selective pressure against the host, positive and negative selection sites in the HuSaV RdRp and VP1 proteins were analyzed and are shown in Supplementary Table S6.

The number of negative selection sites was calculated with three methods (FUBAR, FEL, and SLAC). For the VP1 protein in the GI.1 total strain, Cluster A Cluster B, and GI.2, the predicted negative selection sites were 214, 71, 48, and 37, respectively. On the other hand, the negative selection sites of the RdRp protein were 256, 65, 37, and 45, respectively.

First, the episodic positive selection that occurs on only a subset of evolutionary branches were analyzed using one method (MEME). For the VP1 protein in the GI.1 total strain, Cluster A, Cluster B, and GI.2, the predicted positive selection sites were 4, 1, 1, and 5, respectively. On the other hand, the positive selection sites of the RdRp protein were 2, 1, 0, and 1, respectively. Second, the pervasive positive selection across all the lineages in a phylogenetic tree was detected using three methods (FUBAR, FEL, and SLAC). One residue and three residues in the RdRp protein of the GI.1 total strain were calculated by FEL and FUBAR, respectively. One method (FUBAR) predicted four sites of positive selection in the VP1 protein of the GI.2. However, no positive selection sites were detected in all datasets when calculated by SLAC. Overall, a residue (aa510) in the RdRp protein of the GI.1 total strain, and two residues (aa13, 421) in the VP1 protein of the GI.2 strain were identified as common to episodic and pervasive positive selections. Therefore, these sites are strongly suggested to be under positive selection. Nevertheless, none of these positively selected sites matched the predicted epitope sites.

## 4. Discussion

The purpose of this study was to investigate the molecular evolution of the *RdRp* region and *VP1* gene in HuSaV using various bioinformatics technologies. In particular, this study focused on two HuSaV genotypes, GI.1 and GI.2, which have been the predominant genotypes worldwide [7,9,10,11]. These genotypes are closely related genetically and phylogenetically [36,37]. It is extremely important to compare and comprehensively analyze the *RdRp* region and *VP1* gene of these genotypes. Moreover, the HuSaV GI.1 strains were divided into two clusters and the differences between these clusters were examined in detail.

Similar to NoV, SaV can undergo recombination between the non-structural protein-encoding region (including the *RdRp* region) and the *VP1* gene, resulting in new chimera viruses [1,38]. In fact, the occurrence of recombination in SaVs has been reported to date [39,40,41]. Recombination, as well as evolution, is essential for viral survival. As previously reported, the molecular evolution of NoV may be influenced by the recombination between *RdRp* and *VP1* [14]. In this study, the phylogenetic trees (both ML and time-scaled trees) of the *RdRp* region and *VP1* gene did not find recombination events in HuSaVs GI.1 and GI.2. The *RdRp* region and *VP1* gene may have co-evolved in HuSaV GI.1 and GI.2, respectively.

The evolutionary rates of the *VP1* gene in HuSaV GI.1 and GI.2 were 1.763 and 1.350 × 10^−3^ substitutions/site/year, respectively. The rates for these HuSaV genotypes are likely lower than that of various NoV genotypes such as GII.2 (2.987 × 10^−3^ substitutions/site/year) [14], Gll.3 (4.82 × 10^−3^ substitutions/site/year) [42], GII.4 (7.68 × 10^–3^ substitutions/site/year) [43], GII.6 (3.345 × 10^−3^ substitutions/site/year) [13], and GII.P17-GII.17 (2.7 × 10^−3^ substitutions/site/year) [44]. Therefore, it is possible that the genes of HuSaVs have evolved more slowly than those of the NoVs. To evaluate the evolutionary rates of viral genes, it may be possible to predict the emergence of new variants that are better able to evade host immunity [43]. Thus, NoVs frequently causes global pandemics because the high evolutionary rates permit the NoVs to mutate for immune evasion [45]. On the other hand, SaV, which mutates less frequently than NoV, may not have escaped human herd immunity to date. Consequently, continuous evolutionary analyses of these genes are needed in the future.

As previously reported for NoV, the evolution of the *VP1* gene affects the *RdRp* region [14,46]. In this study, the time-scaled phylogenetic trees suggested that the *RdRp* region and *VP1* gene of HuSaV GI.1 divided into two clusters, Cluster A and Cluster B, around 1969 and 1964, respectively. Moreover, the different evolutionary rates between the two HuSaV GI.1 clusters were estimated. The evolutionary rates of the *VP1* gene were higher in Cluster B (2.168 × 10^−3^ substitutions/site/year) than in Cluster A (1.745 × 10^−3^ substitutions/site/year). Similarly, the rate in the *RdRp* region of Cluster B (3.037 × 10^−3^ substitutions/site/year) was faster than that of Cluster A (3.037 × 10^−3^ substitutions/site/year). The rapid evolutionary rate of Cluster B may be related to the increase in population genome size. These results suggest that Cluster A and B may have evolved independently. Because no recombination between the *RdRp* region and *VP1* gene was observed within SaV GI in this study, the evolutionary differences between clusters are not due to the recombination, as previously reported [14]. Further work is needed to clarify the differences in the evolutionary characteristics of the genotypes and clusters.

The phylogenetic distance analyses estimated that the *RdRp* region and *VP1* gene of HuSaV GI.1 Cluster A were more genetically wide than those of Cluster B (Figure 4). These results suggest that the *RdRp* region and *VP1* gene in Cluster A have high diversity. The possible reason for this diversity may be that HuSaV GI.1 Cluster A has been detected for a long period of time (2001–2020) (Supplementary Figure S1) and has branched into two sub-clusters topologically in the phylogenetic trees (Figure 1, Supplementary Figure S3). On the other hand, the *RdRp* region and *VP1* gene of HuSaV GI.1 Cluster B may be highly conserved, because Cluster B was detected over a short period of time (2011–2017) (Supplementary Figure S1). Nevertheless, a high evolutionary rate and an increase in the effective population sizes were observed in HuSaV Cluster B. Thus, it is possible that the gene of HuSaV Cluster B may be rapidly evolving.

The HuSaV strains diverged from the porcine SaV GIII around 900 BC (Supplementary Figure S4). The domestication of pigs is believed to have started around 9000 to 10,000 years ago [47]. It has been suggested that SaV may have been transmitted from pigs to humans. On the other hand, a previous report suggested that NoV GIII (bovine type) was divided from NoV GII (human type) around 854 CE [48]. Moreover, Group A rotavirus is a zoonotic pathogen that infects a variety of mammalian animals, particularly domestic animals such as cattle and pigs [49]. Domestic animals that come into close contact with humans increase the risk of the cross-species transmission of pathogens, which may potentially cause pandemics [50]. Some genotypes of SaV can infect various animals; thus, SaV is also considered a zoonosis [51]. Since it is possible that SaV from animals may infect humans, it is necessary to study the relationship between genotypes that infect other animal species and HuSaV in the future. Such an approach would contribute to the development of One Health surveillance.

The RdRp diner protein of HuSaV GI.1 had more sites that were strongly predicted as epitopes than that of GI.2. In contrast, the VP1 protein of HuSaV GI.2 had more sites that were strongly predicted as epitopes than GI.1. In particular, the apex of the protrusion of the VP1 protein was strongly predicted as epitopes. Because it has been reported that the VP1 protein of SaV is a target of host immunity [52], it is possible that GI.1 has evolved through escape mutations. Therefore, in the future, a variant that evades host immunity may emerge from genotype GI.1. In particular, Cluster A, which has a rapid evolutionary rate and an increasing genome population size among SaV GI, should be noticeable.

In a recent report, Ji et al. also analyzed the whole genome, focusing on HuSaV GI.1 [53]. They constructed a phylogenetic tree using the whole genome of GI.1 and classified it into two lineages: Lineage I and Lineage II, which correspond to Clusters B and A in this study. In contrast, we analyzed the *RdRp* region and *VP1* gene of HuSaV GI.1 and GI.2, separately. It was found that the two genes have evolved with different characteristics, providing important insights into understanding the function and evolution of these viral proteins. For molecular evolutionary research in viral genes, it is important to analyze individual genes as well as the whole genome sequence.

Similar to NoV, it is important to study the *RdRp* and *VP1* of SaV. However, there is no clear classification definition for the *RdRp* genotype of SaV. Therefore, there may be limitations to the molecular evolution studies and molecular epidemiological surveys focusing on the SaV *RdRp* gene and RdRp protein. As with NoV, an international definition of the *RdRp* classification in SaV is eagerly awaited.

## 5. Conclusions

In conclusion, the most recent common ancestors of the *VP1* genes in HuSaV GI.1 and GI.2 emerged around the 1930s. The evolutionary rate of strains belonging to HuSaV GI.1 Cluster B was significantly higher than that of strains belonging to HuSaV GI.1 Cluster A and GI.2. Moreover, the effective population size of the HuSaV GI.1 Cluster B rapidly increased before 2010. These results suggest that the HuSaV GI.1 Cluster B may be evolving more actively than other genotypes. Furthermore, conformational B-cell epitopes were more strongly predicted in the VP1 protein of GI.2 than in that of GI.1. Together, these findings may contribute to a better understanding of HuSaV virology and molecular evolution.

## Data Availability

The raw data supporting the conclusions of this article will be made available by the authors on request.

## Supplementary Materials

The following supporting information can be downloaded at: https://www.mdpi.com/article/10.3390/microorganisms13020322/s1, Figure S1: Timeline distribution of sequences by collected year in this study; Figure S2: Phylogenetic trees of the *RdRp* and *VP1* sequences of the various SaV genogroups constructed by the ML method; Figure S3: Phylogenetic trees of *RdRp* regions and *VP1* gene of the HuSaV GI.1, and *RdRp* region and *VP1* gene of the HuSaV GI.2 constructed by the ML method; Figure S4: Time-scaled phylogenetic trees of *RdRp* region and *VP1* gene of t the various SaV genogroups constructed by the Bayesian MCMC method; Figure S5: Similarity plot analysis of the *RdRp* region of the HuSaV GI.1, and *RdRp* region and *VP1* genes of the HuSaV GI.2; Table S1: List of HuSaV strains used in this study; Table S2: Parameters used in the Bayesian Markov chain Monte Carlo (MCMC) analyses; Table S3: Evolutionary rates (Substitutions/Site/Year) of the *RdRp* region and *VP1* gene in HuSaV GI.1 and GI.2; Table S4: Putative conformational epitopes for the RdRp proteins of HuSaV; Table S5: Putative conformational epitopes for the VP1 proteins of HuSaV; Table S6: Number of amino acid residues of predicted positive and negative selection sites in HuSaV.
