# Supplementary material for: Molecular Evolutionary Analyses of the RNA-Dependent RNA Polymerase (RdRp) Region and VP1 Gene in Sapovirus GI.1 and GI.2
Source: Microorganisms. 2025 Feb 1;13(2):322. doi: 10.3390/microorganisms13020322 (PMC11858432; doi:10.3390/microorganisms13020322)
Supplement: Supplementary file 1 [file microorganisms-13-00322-s001.zip › SupplementaryFigures.pdf]

Supplementary data

Molecular Evolutionary Analyses of the RNA-Dependent RNA Polymerase (*RdRp*) Region and *VP1* Gene in Sapovirus GI.1 and GI.2

Fuminori Mizukoshi [1*], Ryusuke Kimura [2,3], Tatsuya Shirai [1], Asumi Hirata-Saito [4], Eri Hiraishi [5], Kosuke Murakami [6], Yen Hai Doan [7], Hiroyuki Tsukagoshi [8], Nobuhiro Saruki [8], Takeshi Tsugawa [9], Kana Kidera [10], Yoshiyuki Suzuki [11], Naomi Sakon [12], Kazuhiko Katayama [10], Tsutomu Kageyama [7], Akihide Ryo [1], Hirokazu Kimura [3,5*]

1 Department of Virology III, National Institute of Infectious Diseases, Musashimurayama-shi, Tokyo 208-0011, Japan; mzksh@niid.go.jp (F.M.); shirai@niid.go.jp (T.S.); aryo@niid.go.jp (A.R.)
2 Department of Bacteriology, Graduate School of Medicine, Gunma University, Maebashi-shi 371-8511, Gunma, Japan; m2220015@gunma-u.ac.jp
3 Advanced Medical Science Research Center, Gunma Paz University, Takasaki-shi 370-0006, Gunma, Japan; h-kimura@paz.ac.jp (H.K.)
4 Department of Microbiology, Tochigi Prefectural Institute of Public Health and Environmental Science, Utsunomiya-shi, Tochigi 329-1196, Japan; saitoa01@pref.tochigi.lg.jp
5 Department of Health Science, Gunma Paz University Graduate School of Health Sciences, Takasaki-shi 370-0006, Gunma, Japan; er.hiraishi@gmail.com
6 Center for Emergency Preparedness and Response, National Institute of Infectious Diseases, Shinjuku-ku, Tokyo 162-8640, Japan; ko-mura@niid.go.jp
7 Center for Emergency Preparedness and Response, National Institute of Infectious Diseases, Musashimurayama-shi, Tokyo 208-0011, Japan; yendoan@niid.go.jp (Y.H.D); tkage@niid.go.jp (T.K.)
8 Gunma Prefectural Institute of Public Health and Environmental Sciences, Maebashi-shi 371-0052, Japan; tsuka-hiro@pref.gunma.lg.jp (H.T.); saruki-n@pref.gunma.lg.jp (N.S.)
9 Department of Pediatrics, Sapporo Medical University School of Medicine, Sapporo-shi 060-8543, Japan; tsugawat@sapmed.ac.jp
10 Laboratory of Viral Infection Control, Ōmura Satoshi Memorial Institute, Graduate School of Infection Control Sciences, Kitasato University, 5-9-1, Shirogane, Minato-ku, Tokyo 108-8641, Japan; kidera.kana@st.kitasato-u.ac.jp (Kana Kidera); katayama@lisci.kitasato-u.ac.jp (Kazuhiko Katayama)
11 Division of Biological Science, Department of Information and Basic Science, Graduate School of Sciences, Nagoya City University, Nagoya-shi, Aichi 467-8501, Japan; yossuzuk@nsc.nagoya-cu.ac.jp
12 Department of Microbiology, Osaka Institute of Public Health, Osaka 537-0025, Japan; nsakon@iph.osaka.jp

* Correspondence: mzksh@niid.go.jp; Tel.: +81-425-61-0771 (F.M.); h-kimura@paz.ac.jp; Tel.: +81-27-365-3366 (H.K.)

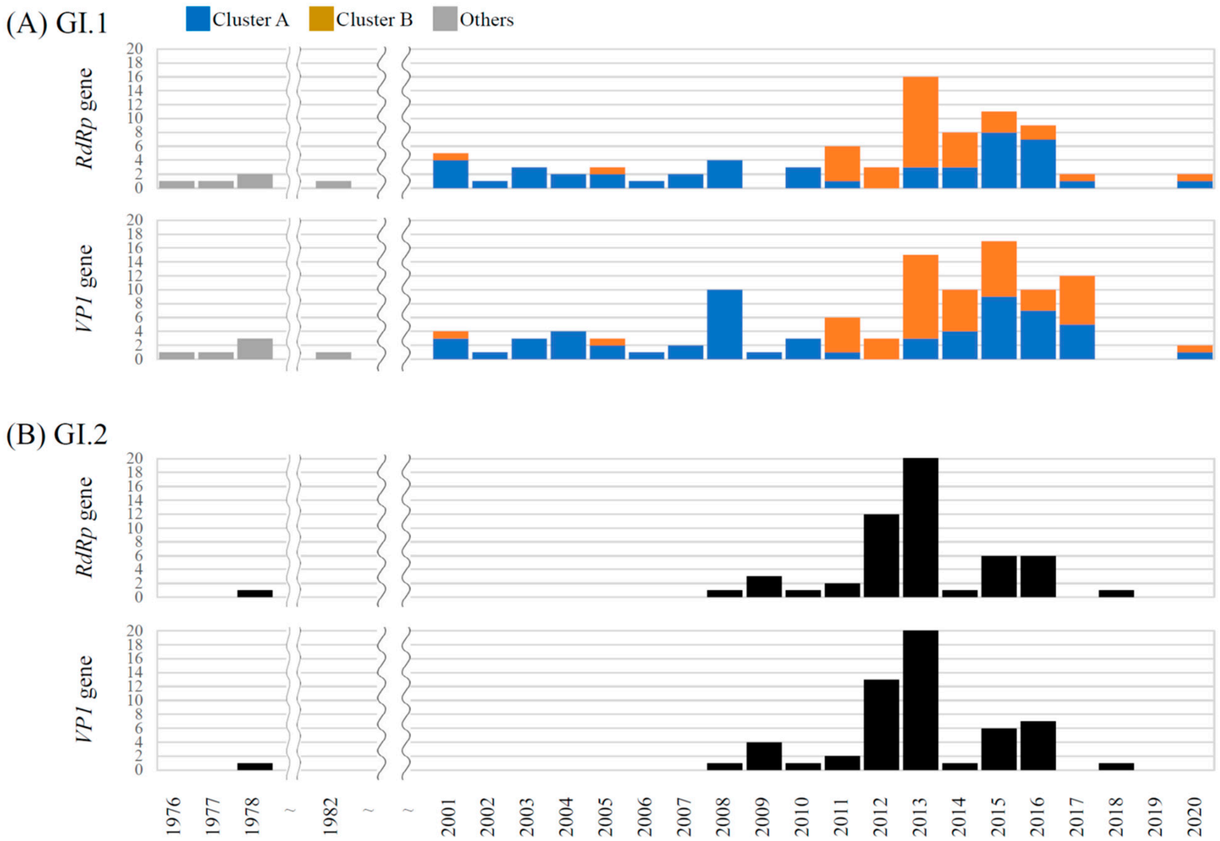

Supplementary Fig S1

Timeline distribution of sequences by collected year in this study. The *RdRp* region and *VP1* gene in HuSaV GI.1 (A) and GI.2 (B) genotypes were shown, respectively.

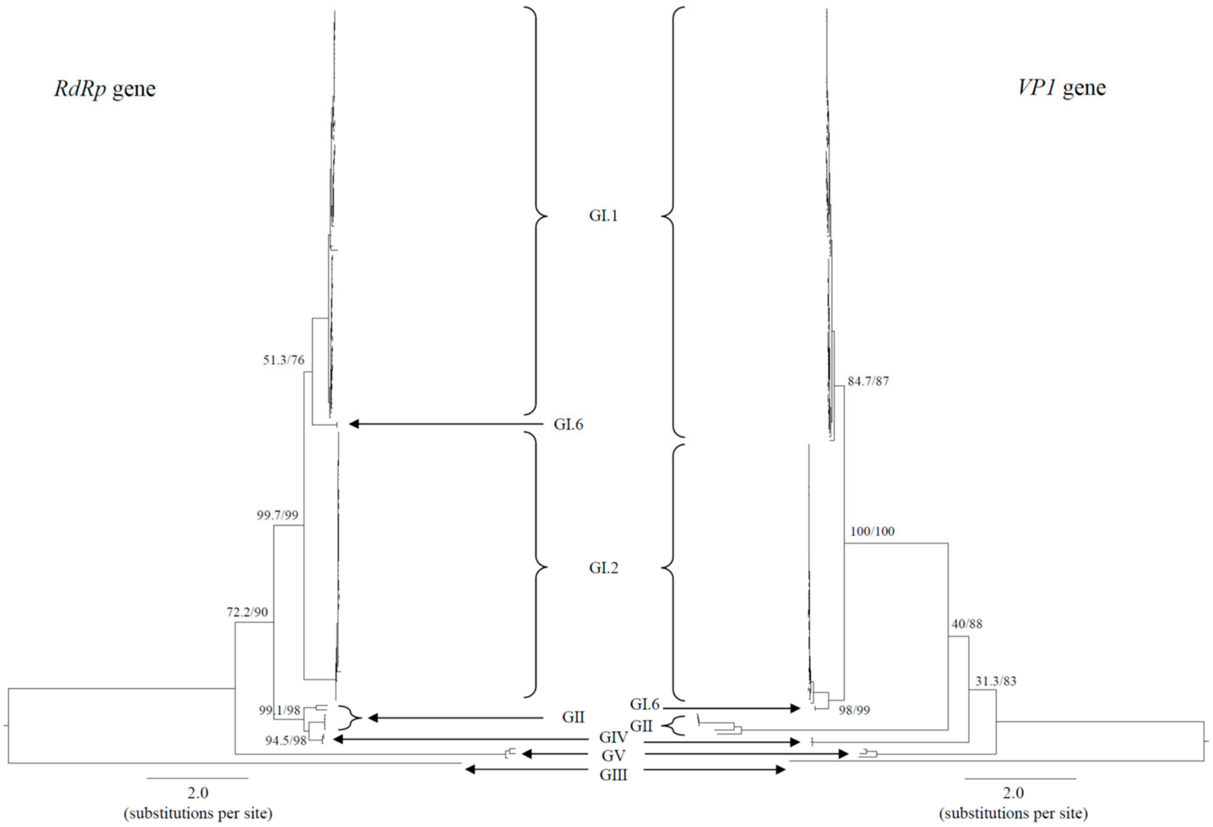

Supplementary Fig S2

Phylogenetic trees of the *RdRp* and *VP1* sequences of the various SaV genogroups (HuSaV GI.1 and GI.2, plus GI.6, GII, GIV, GV, and porcine SaV GIII) constructed by the ML method. Labels at the branch nodes show SH-aLRT / UltrafastBootstrap values. The scale bar shows nucleotide substitutions per site. The substitution model for the *RdRp* and the *VP1* gene was the TIM2+F+G4 model selected with ModelFinder.

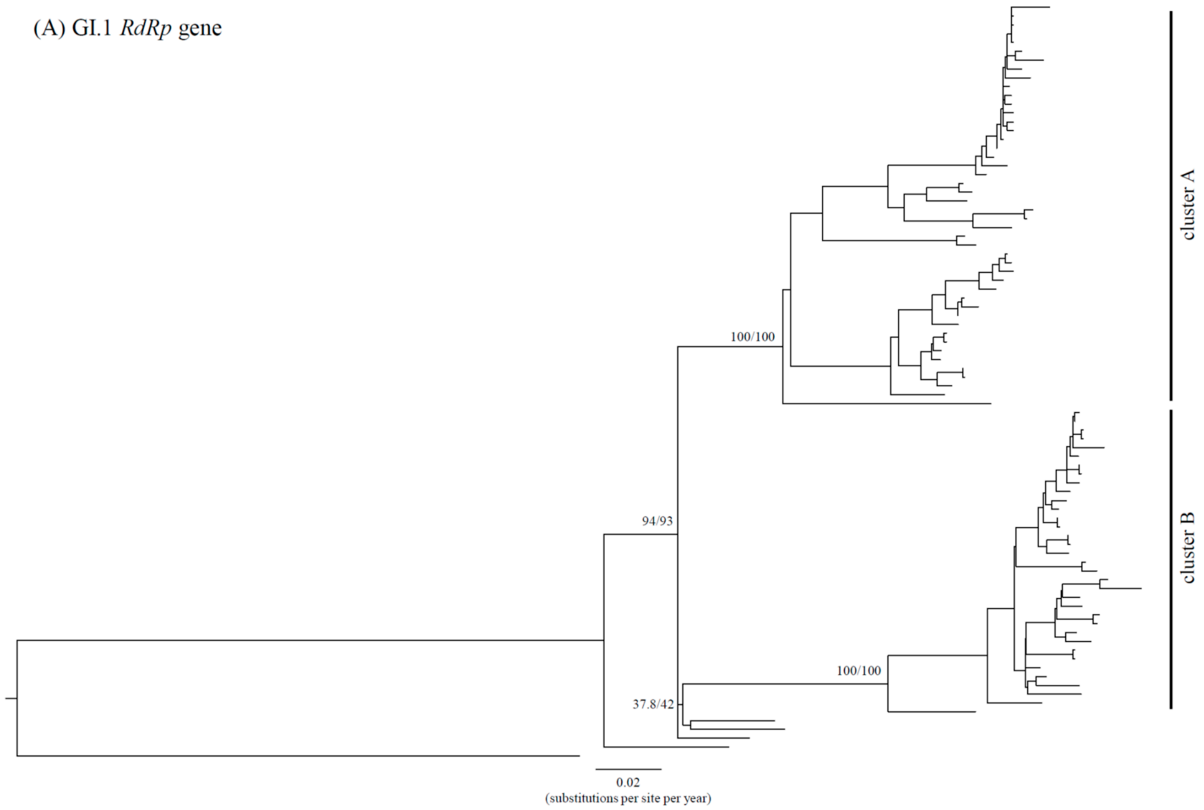
(A) GI.1 *RdRp* gene
100/100
94/93
37.8/42
100/100
cluster A
cluster B
0.02
(substitutions per site per year)

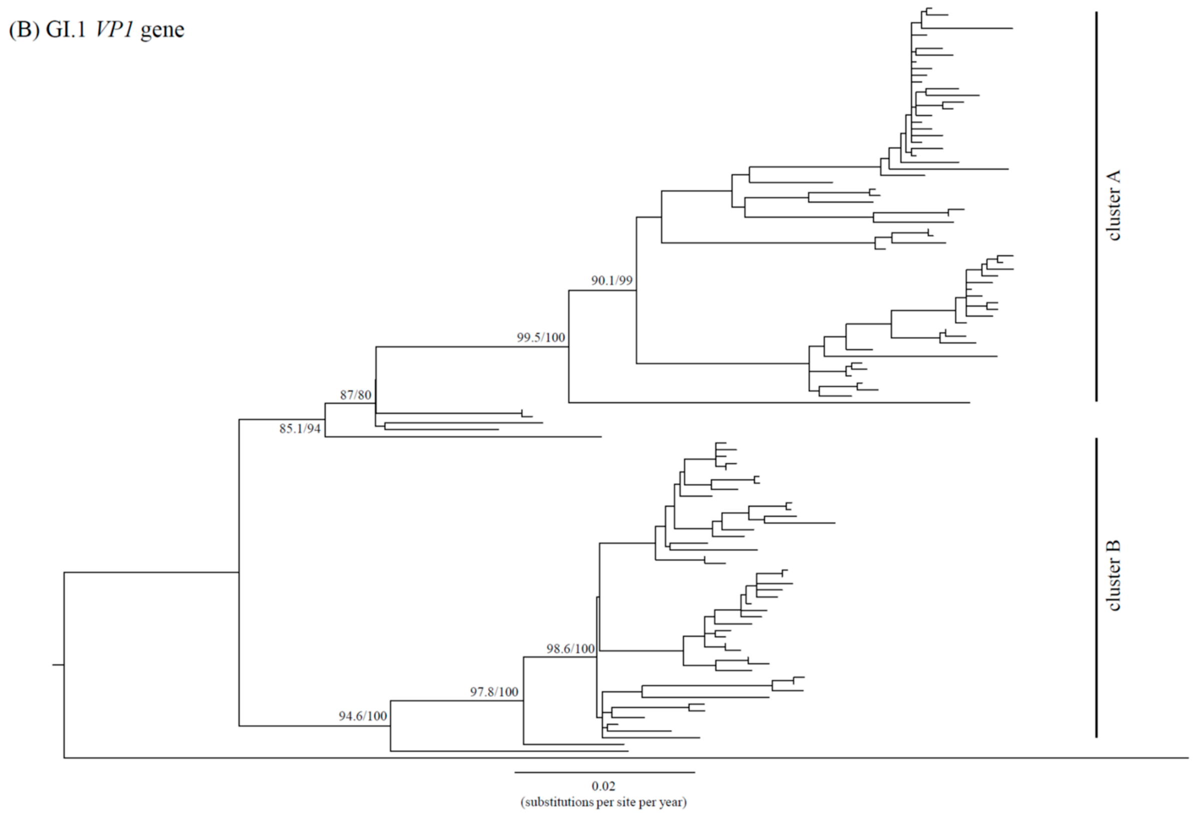
(B) GI.1 *VP1* gene
90.1/99
99.5/100
87/80
85.1/94
98.6/100
97.8/100
94.6/100
cluster A
cluster B
0.02
(substitutions per site per year)

(C) GI.2 *RdRp* gene

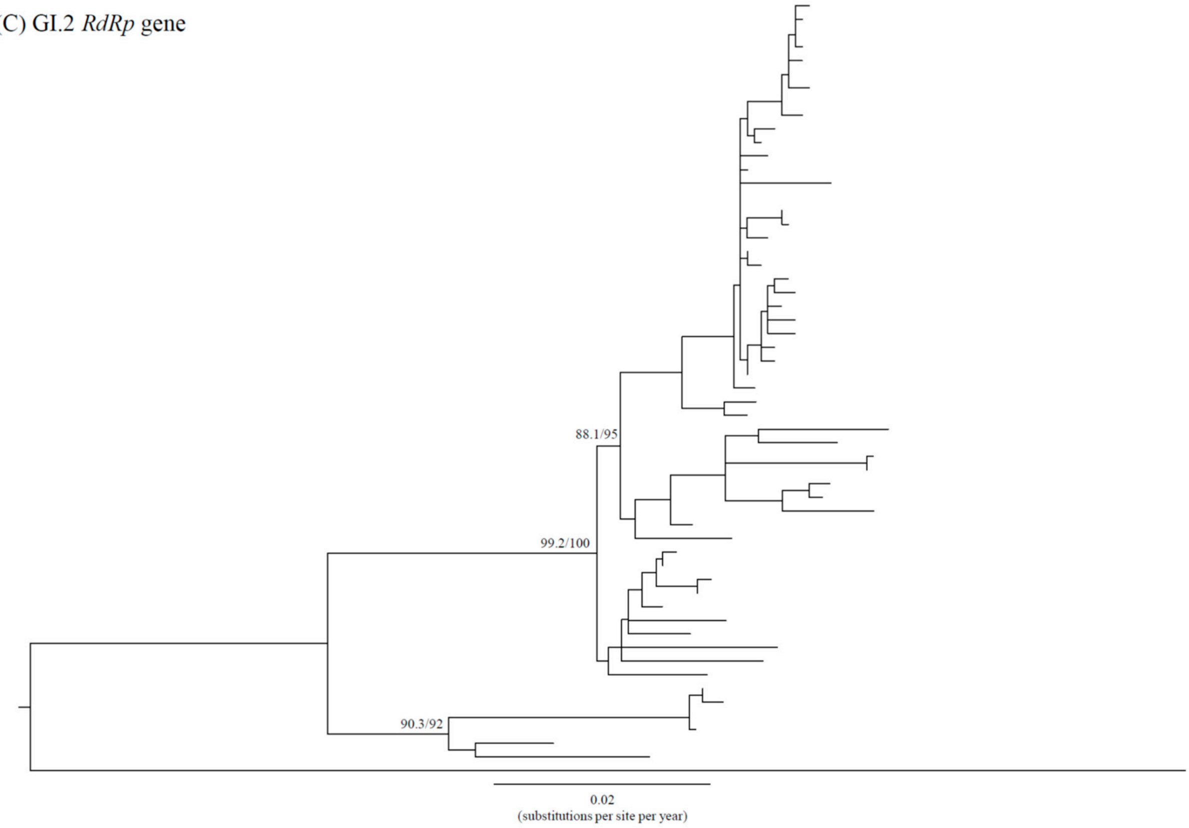

(D) GI.2 *VP1* gene

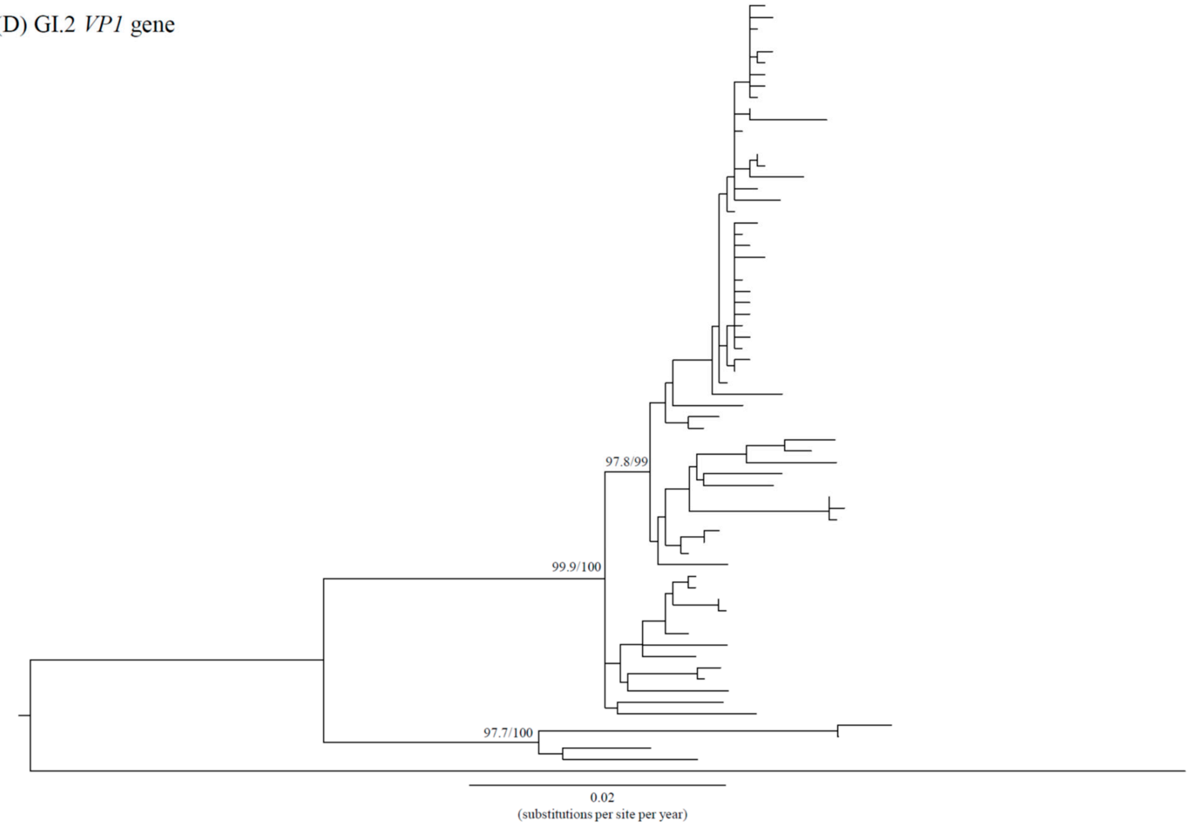

Supplementary Fig S3

Phylogenetic trees of *RdRp* regions (A) and *VP1* gene (B) of the HuSaV GI.1, and *RdRp* region (C) and *VP1* gene (D) of the HuSaV GI.2 constructed by the ML method. Labels at the branch nodes show SH-aLRT / UltrafastBootstrap values. The scale bar shows nucleotide substitutions per site. The substitution models selected with ModelFinder were SYM+I+R2, TIM3e+I+G4, TNe+I, and TIM2e+G4 for RdRp regions (A) and VP1 gene (B) of the HuSaV GI.1, and RdRp region (C) and VP1 gene (D) of the HuSaV GI.2, respectively.

(A) *RdRp* gene

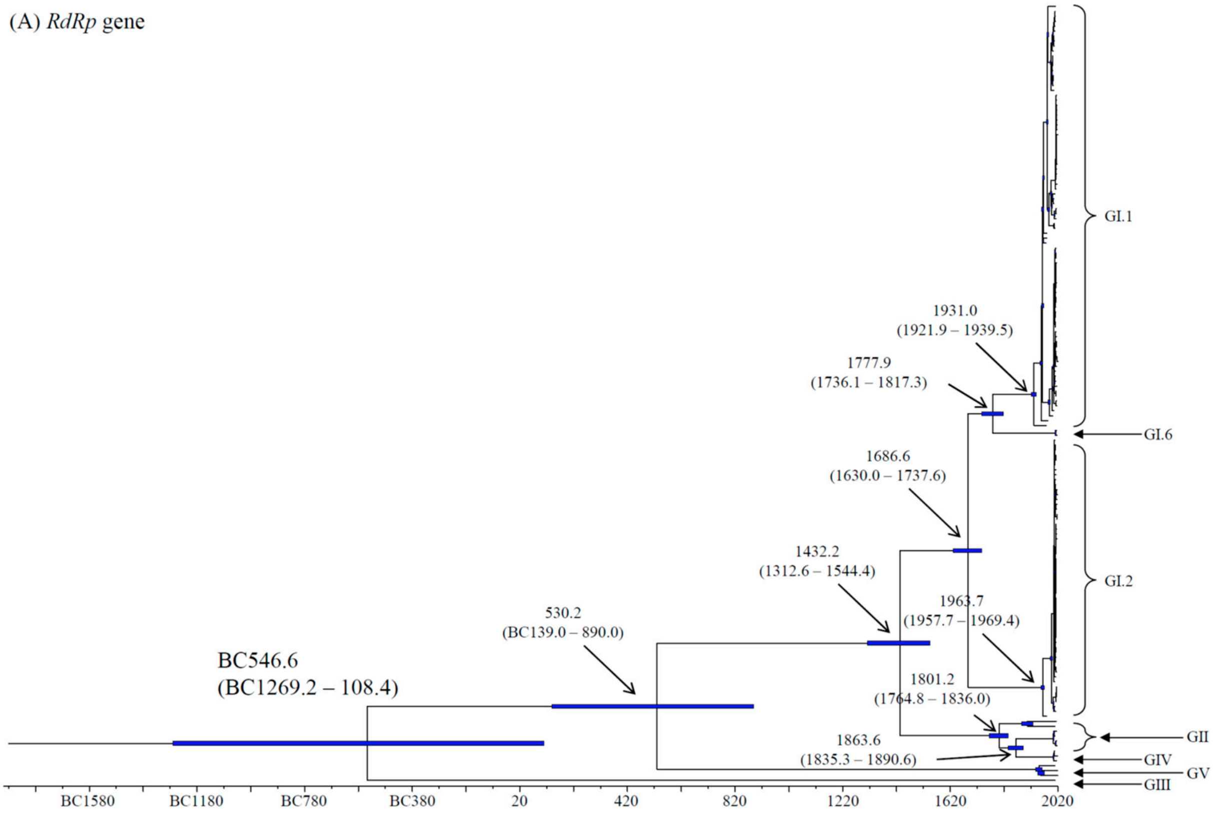

(B) *VP1* gene

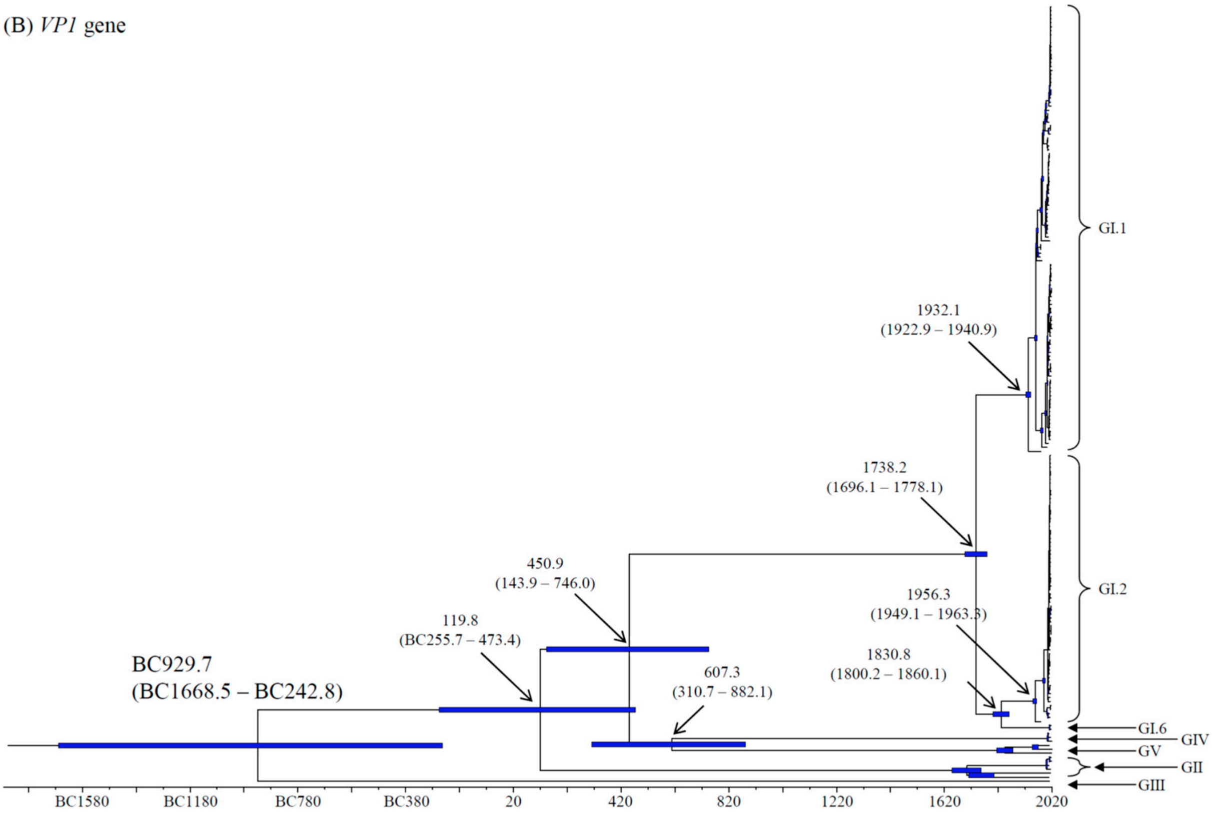

Supplementary Fig S4

Time-scaled phylogenetic trees of *RdRp* region (A) and *VP1* gene (B) of t the various SaV genogroups (HuSaV GI.1 and GI.2, plus GI.6, GII, GIV, GV, and porcine SaV GIII) constructed by the Bayesian MCMC method. The horizontal axis represents time (years). Blue bars indicate the 95% HPD for a branched year.

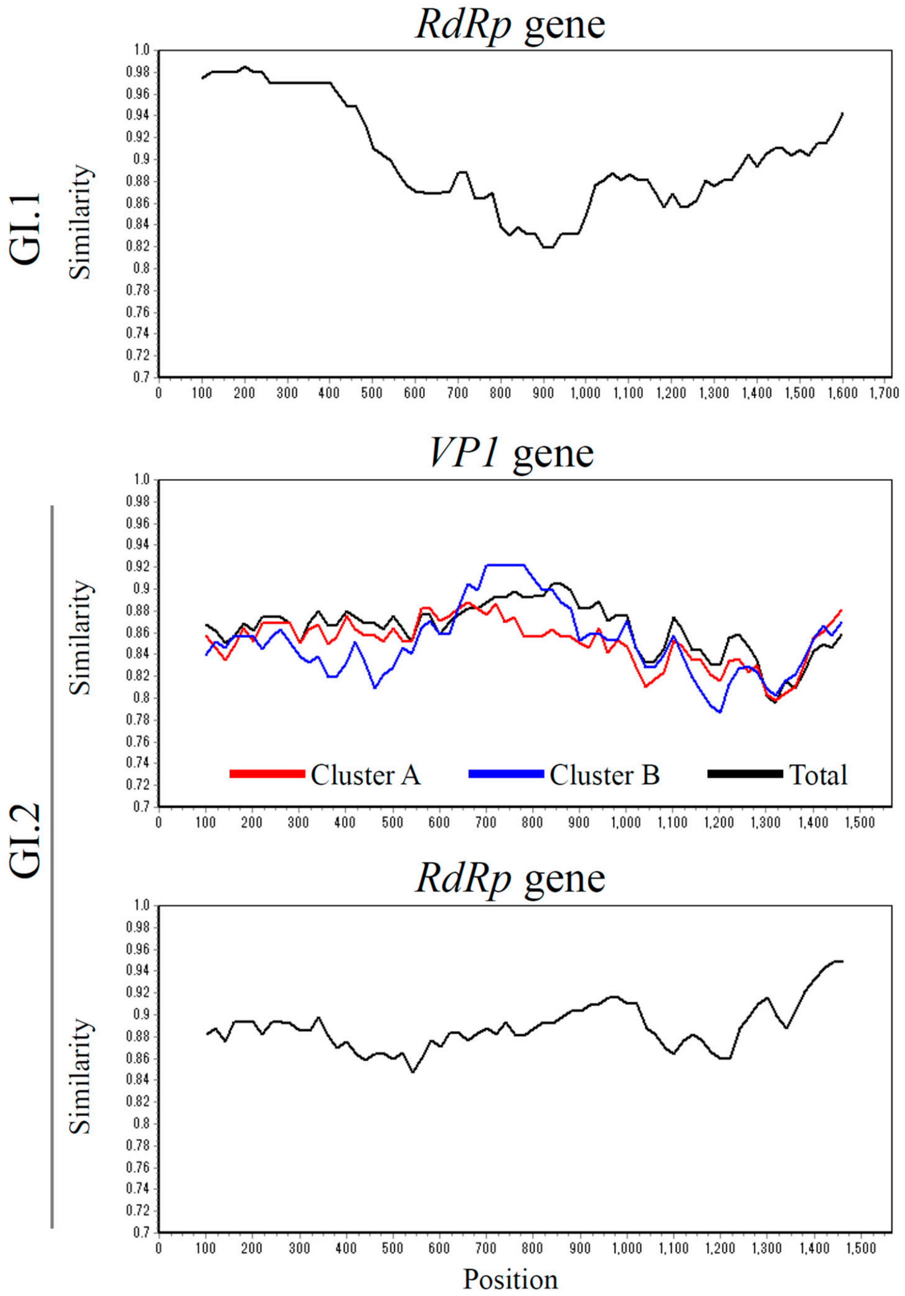

Supplementary Fig S5

Similarity plot analysis of the *RdRp* region of the HuSaV GI.1 (A), and *RdRp* region (B) and *VP1* genes (C) of the HuSaV GI.2. Nucleotide similarity to the prototype strain (GI.1: MN794208, collected in 1977; GI.2: MN794205, collected in 1978) was calculated using SimPlot analysis.
